# *In Vivo* Mapping of Vascular Inflammation Using Multimodal Imaging

**DOI:** 10.1371/journal.pone.0013254

**Published:** 2010-10-11

**Authors:** Benjamin R. Jarrett, Carlos Correa, Kwan Liu Ma, Angelique Y. Louie

**Affiliations:** 1 Department of Biomedical Engineering, University of California Davis, Davis, California, United States of America; 2 Department of Computer Science, University of California Davis, Davis, California, United States of America; Maastricht University, The Netherlands

## Abstract

**Background:**

Plaque vulnerability to rupture has emerged as a critical correlate to risk of adverse coronary events but there is as yet no clinical method to assess plaque stability *in vivo*. In the search to identify biomarkers of vulnerable plaques an association has been found between macrophages and plaque stability—the density and pattern of macrophage localization in lesions is indicative of probability to rupture. In very unstable plaques, macrophages are found in high densities and concentrated in the plaque shoulders. Therefore, the ability to map macrophages in plaques could allow noninvasive assessment of plaque stability. We use a multimodality imaging approach to noninvasively map the distribution of macrophages *in vivo*. The use of multiple modalities allows us to combine the complementary strengths of each modality to better visualize features of interest. Our combined use of Positron Emission Tomography and Magnetic Resonance Imaging (PET/MRI) allows high sensitivity PET screening to identify putative lesions in a whole body view, and high resolution MRI for detailed mapping of biomarker expression in the lesions.

**Methodology/Principal Findings:**

Macromolecular and nanoparticle contrast agents targeted to macrophages were developed and tested in three different mouse and rat models of atherosclerosis in which inflamed vascular plaques form spontaneously and/or are induced by injury. For multimodal detection, the probes were designed to contain gadolinium (T1 MRI) or iron oxide (T2 MRI), and Cu-64 (PET). PET imaging was utilized to identify regions of macrophage accumulation; these regions were further probed by MRI to visualize macrophage distribution at high resolution. In both PET and MR images the probes enhanced contrast at sites of vascular inflammation, but not in normal vessel walls. MRI was able to identify discrete sites of inflammation that were blurred together at the low resolution of PET. Macrophage content in the lesions was confirmed by histology.

**Conclusions/Significance:**

The multimodal imaging approach allowed high-sensitivity and high-resolution mapping of biomarker distribution and may lead to a clinical method to predict plaque probability to rupture.

## Introduction

Atherosclerosis is a progressive disease characterized by the formation of arterial plaques. However, the origin of most acute vascular events is atherothrombosis, the formation of life-threatening clots and it is currently accepted that plaque rupture and erosion are the major causes for atherothrombosis[1]. Vulnerable plaques are defined to be any lesions prone to thrombose [2]. Intense interest in the pathology of vulnerable plaques has lead to the recognition that plaque composition, more than degree of vessel occlusion, is the primary determinant of stability. Plaques prone to rupture are generally characterized by thin fibrous caps (<65 mm), large, lipid-rich cores, with high macrophage content (>25 macrophages/3 mm diameter field of view)[2], [3], [4], [5], [6]. Macrophages present in the developing plaque release cytokines and other factors that can weaken the fibrous cap, eventually leading to plaque instability and rupture[7], [8], [9].

In the coronary arteries, numerous reports have observed that high macrophage density is characteristic of lesions vulnerable to rupture [3], [4], [5], [9], [10], [11]. Furthermore, it has been observed that the pattern of distribution of macrophages in the plaque correlates with degree of instability. An ex vivo study of human coronary artery plaque specimens showed that the extent of inflammation at the plaque shoulders appears to correlate with degree of vulnerability—slightly unstable plaques have little or no inflammation at the plaque shoulders, while highly unstable plaques have extensive inflammation at the plaque shoulders [10]. Therefore, the ability to image plaques at high resolution to determine macrophage content and distribution could provide a means to noninvasively assess plaque vulnerability and degree of risk to rupture in inflamed arteries. There is currently no clinical method to assess plaque vulnerability *in vivo*; the ability to do so could provide a critical diagnostic to guide management of patients with vascular disease.

The gold standard for imaging atherosclerotic disease is angiography. Angiographic images provide information on decreasing of vessel lumen as plaques invade the luminal space. Highly stenotic plaques may be revealed by this technique; however angiography cannot provide direct assessment of the extent of disease in the vessel wall, nor can it detect disease in vessels that have positive remodeling to enlarge vessel diameter in response to plaque growth. The recognition that the majority of clots leading to acute coronary events occur in plaques that are not highly stenotic [12] highlighted the need for alternative imaging methods that can directly image the vessel wall.

There are a number of alternative techniques to image plaques including invasive modalities such as intravascular ultrasound, angioscopy, thermography, optical coherence tomography, raman spectroscopy, near infra-red spectroscopy and intravascular MRI[12], [13], [14], [15]. These invasive techniques involve intravascular transceivers that must be threaded into the vessel being examined and therefore are unsuitable for exploratory imaging to assess overall plaque burden in the patient. Noninvasive methods are better suited for examining larger regions; ultrasound, computed tomography and magnetic resonance imaging have received the most attention[16], [17]. Ultrasound and computed tomography can provide information about cap thickness and plaque calcification but MRI shows the most promise for assessing both structure and lipid composition to evaluate plaque stability[12], [16], [18]. However, MRI lacks the sensitivity to screen large regions and atherosclerotic disease can occur anywhere in the vascular system. Therefore, we have developed multimodal imaging methods to screen for inflamed lesions with high sensitivity using PET and visualize macrophage localization in plaques at high resolution using MRI.

## Methods

### Ethics Statement

All animal experiments were performed under a protocol approved by the UC Davis Institutional Animal Care and Use Committee (approved protocol #12115). Anaesthesia was administered for all imaging and surgical procedures. Post-procedural analgesics were given post-surgery and as needed. The animals were monitored regularly for pain or discomfort.

### Synthesis of copper-64 (^64^Cu) labeled dextran sulfate coated iron oxide nanoparticles

Dextran coated particles were synthesized as described previously[19], [20]; the dextran coated particles were then cross-linked and aminated[21], [22]. Detailed methods and source information are provided in Materials and Methods S1. Briefly, cross-linked iron oxides (CLIO) were synthesized by adding epichlorohydrin (510 molar equivalents to Fe) to an aqueous solution of dextran coated particles in 2.5 M NaOH (0.5 mol NaOH:12 mmol Fe), and stirring at room temperature (RT) over night. CLIO was purified by dialysis against nanopure water, then aminated by addition of ammonium hydroxide (NH_4_OH, 28–30%) in a 10∶1 (volume:volume) NH_4_OH/particle solution. This solution was stirred overnight at RT and then purified by dialysis. The purified product was then filtered with 0.2 µm pore membranes to yield a black, translucent solution.

Radiolabeling of the dextran particles was achieved by first mixing ^64^CuCl_2_ in 10 mM HCl with 1 M triethanolamine acetate to form a ^64^Cu-OAc complex in a 0.1 M TEAA solution with a pH of 7.0 (6 µL, 2.84 mCi^64^CuOAc used for reaction). The copper-64 acetate solution was mixed with *p*-SCN-Bz-DOTA (p-benzyl isothiocyanate-1,4,7,10-tetraazacyclododecane-1,4,7, 10-tetraacetic acid, 0.2 µmol, 10 µL) at 55°C for 30 min. *p*-SCN-Bz-DOTA(^64^Cu) (2.84 mCi) was then reacted with CLIO-NH_2_ nanoparticles (150 µL, 0.1 M TEAA (triethyl ammonium acetate), pH 7, 28.7 µmol Fe) at 55°C for 60 min. The nanoparticles were purified by size exclusion chromatography (SEC) on a Sephadex G25 column equilibrated with 0.9% NaCl using centrifugation (2000 rpm, 2 min). This was repeated 3 times to yield CLIO-DOTA(^64^Cu).

### Synthesis of gadolinium/^64^Cu labeled maleylated bovine serum albumin

Copper-64 labeling of maleylated bovine serum albumin (Mal-BSA) was performed as described previously[23] and is described in detail in Materials and Methods S1.

### *In vivo* MRI/PET

All animal experiments were performed under a protocol approved by the UC Davis International Animal Care and Use Committee. The temperature inside the coil where the animal was placed was maintained at 37°C, electrocardiogram (ECG) and respiration were monitored (MP150, Biopac, Goleta, CA). During imaging the animals were anesthetized by isoflurane inhalation (2% in 100% oxygen, IsoFlo; Abbott Laboratories).

### Rat imaging

Sprague Dawley rats (100–125 g, Charles River Laboratories, Wilmington, MA) were used as a mechanical injury model of vascular inflammation. Two models were used, (1) a carotid crush injury of the carotid, or (2) implantation of a copper cuff.

For the crush injury, rats (n = 7) were anaesthetized by ketamine/xylazine (88 mg/kg and 2.5 mg/kg) and approximately 1 cm of the common carotid artery was isolated from the carotid sheet and the carotid artery was crushed with tweezers for 10 seconds. One week was allowed for progression of inflammation and recruitment of macrophages, as it has been shown that inflammatory events at the endothelial layer (e.g. expression of inflammatory cell adhesion molecules E-selectin and P-selectin) begin as early as one hour after induction of the injury[24].

For the copper cuff model, rats (n = 4) were anaesthetized by ketamine/xylazine (75–100 mg/kg and 5 mg/kg) and a silicone-copper cuff was implanted around the right carotid artery[25]. The copper cuffs were prepared by looping copper wire (0.1 mm) 3 to 4 times around a 1.8 mm mold and embedding in a thin layer silicone. After curing, the cuffs were cut longitudinally and carefully peeled away and sterilized for implantation. The total size of each cuff was about 5 to 6 mm in length and 4 mm in diameter. For implantation, approximately 1.5 cm of the right common carotid artery was isolated from the carotid sheet and the copper cuff carefully place around the vessel before closing the wound site. Up to three weeks were allowed for progression of inflammation and recruitment of macrophages before imaging, as it was shown that inflammatory events in the vessel wall after copper cuff implantation progress over three to 42 days[25].

For proof of principle that PET/MR imaging can reveal plaques using scavenger targeted probes, a maleylated-BSA/DOTA(gadolinium/copper-64) contrast was used[26]. One week following the crush injury, or at one (n = 4), two (n = 3), and three (n = 2) weeks after implantation of the copper cuff the dual MRI/PET contrast agent was administered; 400–800 µCi of copper was injected via the tail vein. As a control, uninjured Sprague Dawley rats (n = 3) were injected with 400–500 µCi of the maleylated-BSA/DOTA(Gd/^64^Cu) via tail vein.

24 hours after contrast agent injection the rats were anesthetized with isoflurane and scanned with a custom built microPETII[27] and then imaged by MRI using a 7T BioSpec (Bruker, Billerica, MA). For the PET imaging, the axial and transaxial field-of views (FOVs) were 4.9 cm and 8.5 cm, with an energy window of 250–750 keV, a timing window of 6 ns, a scan time of 60 minutes, and a fully 3D *maximum a posterioi* (MAP) reconstruction was performed resulting in a spatial resolution of 0.4×0.4×0.58 mm^3^ for a 128×128×83 matrix[28]. For MR imaging, a respiratory gated T1 weighted FLASH sequence was used with repetition time TR  = 690 ms, echo time TE  = 3.7 ms, flip angle  = 45°, 59 slices, 0.75 mm slice thickness, a FOV  = 5.5×4.0 cm^2^, and a matrix of 512×256 resulted in an in-plane resolution of 0.107×0.156 mm^2^. Three fiducial markers (glass bulbs filled with an aqueous solution of ∼0.5 µCi ^64^Cu each) were positioned on the animal and used as land markers to co-register the MRI and PET data.

### Mouse imaging

ApoE^−/−^ mice (n = 3), average age 13 weeks (Jax West Laboratories, West Sacramento, CA), underwent the carotid artery ligation and after ligation of the right carotid artery were placed on a high cholesterol diet (20.1% fat, 1.25% cholesterol Harlan Teklad, TD.02028) for two weeks prior to imaging using separate PET and MRI scanners; 24 hours after contrast agent injection (18–20 mg Fe/kg and 20–25 uCi/g). the mice were anesthetized with isoflurane and scanned with a custom built microPETII [27] and imaged by MRI using a 7T BioSpec (Bruker, Billerica, MA). For the PET imaging, the axial and transaxial FOVs were 4.9 cm and 8.5 cm, with an energy window of 250–750 keV, a timing window of 6 ns, a scan time of 60 minutes, and a fully 3D *maximum a posterioi* (MAP) reconstruction was performed resulting in a spatial resolution of 0.4×0.4×0.58 mm^3^ for a 128×128×83 matrix[28]. For MR imaging, a respiratory gated T2* weighted Fast Low Angle SHot (FLASH) sequence was used with TR  = 540 ms, TE  = 5 ms, flip angle  = 30°, 40 slices, 0.5 mm slice thickness, a FOV  = 3.5×3.5 cm^2^, and a matrix of 256×256 resulted in an in-plane resolution of 0.137×0.137 mm^2^. Three fiducial markers (glass bulbs filled with an aqueous solution of ∼0.5 µCi ^64^Cu each) were positioned on the animal and used as land markers to co-register the MRI and PET data.

### Image Analysis

For both rat and mouse image data sets, MRI and PET data were analyzed on a commercial 64bit PC workstation. The MRI and PET data coregistered and three-dimensional (3D) volumes corresponding to probe uptake (PET) and vasculature (MRI) were traced manually in the axial, coronal, and sagittal planes using Amira 5 (Mercury Computer Systems, Visage Imaging, Carlsbad, CA). Alternatively, the MRI and PET data was exported into Amide version 0.8.22 (Medical Data Image Analyzer) and co-registered. Signal to noise ratio (SNR) and contrast to noise ratio (CNR) of the raw MRI and PET data were determined using ImageJ (National Institutes of Health, version 1.38x). For the MRI data, CNR was defined as abs[Signal_injury_-Signal_tissue_]/Signal_noise_, where the absolute value of the signals is taken to obtain a positive CNR. In the CNR measurements, the regions of interest (ROI) used were the same size for all areas and the normal tissue signal used for contrast determination was the adjacent vessel tissue to the probe uptake. Regions of interest were selected manually on individual slices that comprise the 3D data set. For vessel structure the ROI were selected based on user perception of the anatomical boundries of vessel or valve walls. For probe localization, the ROI were selected based on user perception of contrast between pixels. Details of the methods for three-dimensional rendering, which did not use segmentation for probe localization, are provided in Materials and Methods S1.

### Histology

To confirm the presence of macrophages, tissue samples were prepared for immunohistochemistry following the imaging experiments. After the 24 hour imaging point, the animals were euthanized and the vasculature was rinsed with heparinized saline via cardiac perfusion. The vessels were then isolated and placed in 10% buffered formalin containing 0.2% trypsin (for membrane permiabilization, necessary for antibody treatment). The copper cuff was carefully removed from the artery before removing the artery from the animal and placing in fixative. Tissue was put into fixative for 8 hours at 4°C before transferring to 70% ethanol, and stored in ethanol at 4°C until all radioactive decayed before embedding. Samples were embedded in paraffin and 4 micron thick slices were obtained. Samples were then deparaffinized, rehydrated, and then antigen retrieval was performed by heating the samples in 10 mM citrate buffer (pH 6) for 1 hour in a steam chamber, then incubating the samples (still in the citrate buffer) at 4°C overnight.

After antigen retrieval, endogenous enzyme activity was blocked with peroxide (10 min, RT, 0.3% H_2_O_2_/methanol), followed by non-specific blocking with BSA (1.2 mg/mL, 4°C overnight). Samples were then incubated with the macrophage specific primary antibody anti-CD68 (Serotec, Oxford, UK) at a 1∶100 dilution overnight at 4°C (rat anti-mouse CD68 for apoE^−/−^ mice or mouse anti-rat CD68 for the copper cuff rats). For the rats, a goat anti-mouse secondary antibody coupled to dyelight647 (Serotec) was then incubated at a 1∶100 dilution for 1.5 hours at RT and counterstained with DAPI (300 nM, 5 min). Then a glass cover slip was mounted with Vectashield H-1000 mounting medium (Vector Laboratory, Burlingame, CA), and samples were imaged by fluorescent microscopy on a Nikon Eclipse TE2000-S scope using a 100x (Nikon PlanApo, NA1.4) oil immersion lens. A Nikon mercury arc lamp was used to excite samples with chroma dichroic filter sets for DAPI (Ca^2+^ 380 nm excitation pass), BSA-TAMRA (FITC/TRITC bandpass), and goat anti-mouse IgG-Dyelight647 (FITC/CY5 bandpass).

For the apoE^−/−^ mice a rabbit anti-rat secondary antibody coupled to horseradish peroxidase (HRP, Serotec) was incubated with the samples at a 1∶100 dilution for 1.5 hours at RT. The bound antibody was visualized by counterstaining with diaminobenzidine (DAB); samples were incubated for 10 min at RT in a glass jar with 200 ml of Tris-HCl buffer (pH, 7.5) containing 40 mg DAB and 34 mL 30% H_2_O_2_. After thurough washing to remove excess DAB, the slides were counterstained for iron using the Perl's Prussian blue stain. A coverslip was mounted (Vectashield) and images were acquired with a Canon Cybershot MPEG Movie EX 5.0 megapixel camera with a Scopetronix Maxview 40 plus adapter attached to a Olympus BX51 scope using a 10x (UPlanFluor, 0.3 NA) or 60x (PlanApo, 1.4 NA) oil immersion objective.

## Results and Discussion

Positive contrast macromolecular and negative contrast nanoparticulate multimodal probes were synthesized that are targeted to macrophages through the macrophage scavenger receptor A type 1 (SRA), a cell surface receptor found on macrophages [20], [26]. SRA are highly expressed by activated macrophages but are not otherwise present in normal blood vessels as discussed in our previous works [19], [20], [26]. Two classes of probes were developed: 1. Macromolecular probes loaded with gadolinium (MRI), ^64^Cu (PET), and fluorescent dye (TAMRA, Molecular Probes). 2. Iron oxide nanoparticles loaded with ^64^Cu. The first class of probes provides positive contrast in MR images and is targeted to macrophages by using the SRA ligand maleylated bovine serum albumin (Mal-BSA) as the macromolecule carrier for conjugation of imaging probes, while the second provides negative MRI contrast and is nonspecifically taken up by macrophages. Both also are labeled for detection by PET imaging. We synthesized both T1 and T2 agents to evaluate the ability for each type of probe to visualize inflammation at high resolution in living systems. These probes were utilized for multimodality imaging of vascular plaques in animal models of vascular inflammation to demonstrate the feasibility of using PET screening to guide selection of volumes for high resolution MRI in order to map macrophage distributions in arterial lesions.

While no single animal model ideally recreates the human condition, a number of models have shown sufficient parallel to the human disease to justify their use as models for specific disease traits. The difficulty with modeling vulnerable plaques has been that although stenotic lesions can be induced in animal models, they have not been observed to spontaneously rupture as human lesions do [29]. Recent observations of ApoE knockout mice has indicated that in this model, plaques can and do spontaneously rupture [30], [31], [32]. This knockout mouse also has been observed to form lesions similar to the vulnerable human case which are rich in macrophage content [33], [34]. While this model can produce plaques with unstable phenotype, we found it to be inconsistent, producing macrophage-rich plaques in only a fraction of examined animals. Macrophage laden plaques were more reliably produced if a carotid artery was ligated. We used the ApoE ligation model, therefore, as one of the testbeds for our probes.

### MRI/PET with multimodal negative contrast MRI probes

**Figure 1:**
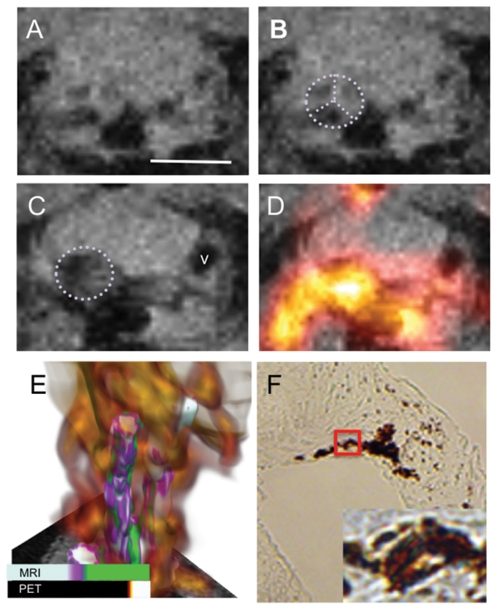
Multimodal nanoparticle probes are taken up by macrophages in the aortic valve of ApoE^−/−^ mice with vascular inflammation induced by ligation. (A) The aortic valve before administration of probes. Scale bar  = 2.5 mm. (B) Position of the aortic valve is indicated by the dotted purple lines. The three leaflets of the valve are clearly evident. (C) The MR signal intensity in and around the aortic valve (dotted outline) decreases after introduction of the multimodal PET/T2-MRI probes, shown 24 h post injection. (D) Coregistration of the PET data with the MRI data illustrates that the PET signal correlates with a broad region including and around the aortic valve. In A–D to determine the grayscale intensity of each pixel in the image and the color intensity of the PET signal, we use bilinear interpolation between the four nearest voxels in the slice. This resampling provides a smoother reconstruction without excessive blurring or loss of data. (E) 3D reconstruction of MRI and PET data shows that the PET signal intensity is a diffuse cloud (orange-yellow) broadly localized around the aortic arch and carotid arteries. This 3D image was generated by combining three different rendering models; details provided in Materials and Methods S1. F) Immunohistochemistry demonstrates that the nanoparticulate probes are localized to macrophages in the aortic arch. Macrophage presence in the aortic valve leaflets is confirmed by positive HRP reaction with DAB, macrophages  =  brown stain (10X magnification). The region in the red box is shown at higher magnification in the inset image (60X) and demonstrates that the iron oxide particles, stained blue, overlap with regions staining for macrophages (brown).

Macrophage targeted multimodal PET/MRI probes were synthesized based on iron oxide nanoparticles as previously described that were targeted to macrophages by coating them with dextran; ^64^CuDOTA was then conjugated to the dextran surface (CLIO-DOTA(^64^Cu))[19], [20]. Materials and methods are available in Materials and Methods S1. ApoE^−/−^ mice that had undergone carotid ligation (n = 3) were imaged 24 hours post injection of dextran coated iron oxide nanoparticles, CLIO-DOTA(^64^Cu). Inflamed lesions were found in all animals and a representative animal is shown in Figure 1.

Temporally, the PET data was acquired first and used to guide volume selection for the MRI images shown in A–D. Fig. 1A shows the aortic arch in an axial MR slice (the 3 valve leaflets can be seen, outlined in Fig. 1B) before administration of CLIO-DOTA(^64^Cu). Twenty four hours post injection of the iron oxide agent the MR signal intensity is observed to decrease, which is attributed to accumulation of iron oxide particles in this region, Fig. 1C, circled area; the contrast-to-noise ratio (CNR) at the aortic valve increased by 68±10%. Upon dissection, the valve itself was observed to be black with accumulated particles. In Fig. 1C the darkened region on the right (v) corresponds to the vena cava, which was further out of the plane of view in panel 1B. Videos including the slices above and below this plane are provided as Videos S1, S2 to confirm that the postinjection contrast changes occurred around the valve and that other apparent contrast differences were due to structures that appear in the plane of view for 1C that were not in the plane for 1B (due to animal position).

Coregistration of the PET signal with the MRI signal at 24 hours, Fig. 1D, confirms that the PET signal correlates with the change in MR signal, verifying localization of the multimodal agent at aortic valve. The three dimensional rendering shown in Fig. 1E reveals the typical relationship between the PET and MRI data where in PET signal, due to lower resolution capability and partial volume effects, is a diffuse cloud over a relatively large region encompassing the carotids while MRI shows discrete maps of macrophage localization. Video version of 1E available in Video S3. It is evident in 1E that the exact anatomical location of probe accumulation would have been difficult to discern from PET alone; indeed, it is difficult to distinguish which artery carries the lesion(s).

Labeling of the aortic valve is consistent with a recent MRI study on apoE^−/−^ mice that were imaged after administration of dextran coated iron oxide particles where particle uptake was seen at the aortic root 48 hours post injection of 30 mg Fe/kg iron oxide particles [35]. These images support that PET guided selection of volumes allows high resolution MRI visualization of small cardiovascular features, such as the aortic valve in living systems. The valve shown is ∼1.5 mm diameter and the three leaflets are clearly resolved.

To further support that the changes in MR and PET signal intensities were due to accumulation of the particles at sites of vascular inflammation, 4 micron sections of the aortic valve were challenged with anti-CD68 monoclonal antibodies, a macrophage marker, and Perl's Prussian blue for iron. Fig. 1F demonstrates that macrophages were present at the aortic valve (the brown stain from the secondary antibody conjugated to HRP, original magnification  = 10X). In the higher magnification image (inset Fig. 1F) one can discern that the iron oxide particles, stained blue, colocalized with macrophages (brown stain).

### MRI/PET of with multimodal positive contrast MRI probes

**Figure 2:**
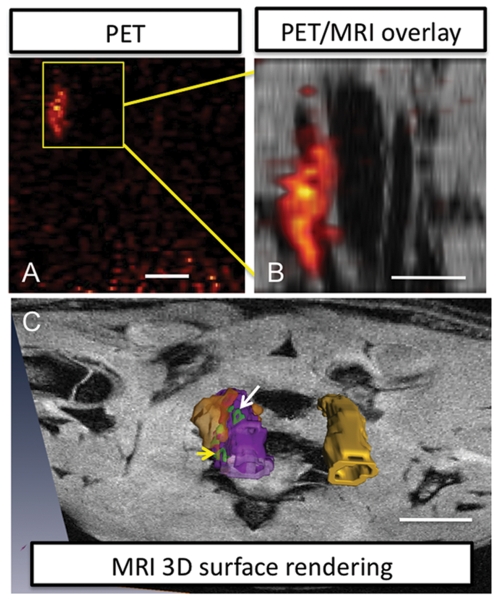
Multimodal macromolecular probes accumulate in the injured vessel of the rat carotid clamp injury model. (A) Coronal view PET image of the rat thorax shows a region of high signal intensity indicating probe accumulation in macrophages, however it is difficult to interpret the tissue of origin for the signal without anatomical information. Scale bar  = 20 mm (B) “Zoomed in” MR images for the volume indicated by the boxed area in A reveal that the PET signal correlates to the carotid artery on the left. Scale bar  = 5 mm. This vessel also shows elevated MR contrast and thicker vessel walls compared to the vessel on the right, (C) 3D reconstruction of MRI and PET data from the carotids is shown protruding from a plane in the MR image. This view illustrates that the probes are localized to the vessel wall of the injured carotid artery (purple), MR signal intensities elevated relative to vessel background signal are rendered in green, and PET intensities are in orange. The contralateral uninjured vessel is yellow. Images rendered by segmentation. MRI high contrast region, in green donut shape, at white arrow is 0.6×1.8 mm (volume is 0.4×2.6×0.6 mm^3^; volume includes regions out of view in this 2D image). MRI high contrast signal at yellow arrow, in green horseshoe shape, is 0.8×1.2 mm, each arm is ∼0.2 mm wide (volume is 1×0.22×3.8 mm^3^). PET volume (orange) is 4.5×8.3×4.0 mm^3^. Scale bar  = 5 mm.

To validate that these effects were not animal-model-specific, nor imaging-method-dependent we also examined rat models of vascular inflammation using the multimodal macromolecular probes (positive MRI contrast). We utilized both the crush[24] injury and copper cuff[25] models, to ensure that our methods could label inflamed plaques regardless of model. The multimodal probes were synthesized as previously described and consisted of ^64^CuDOTA, Gd^3+^ DOTA, and TAMRA-labeled maleylated bovine serum albumin (^64^Cu/Gd/TAMRA-mal-BSA). [23] Rats with unilateral carotid crush injuries were imaged 24 hours after injection of the multimodal probes (number of animals  = 7). A representative animal is shown is Figure 2. Fig. 2 demonstrates uptake of the MRI/PET probe in plaques resulting from carotid crush injury. The PET image clearly shows an accumulation of contrast agent (Fig. 2A), but without anatomical reference it is difficult to determine the tissue of origin. A high resolution MR image that is zoomed in on the volume enhanced in the PET image confirms that the PET signal overlaps with a broad area in and around the injured carotid artery on the left (Fig. 2B). No PET signal is associated with the uninjured contralateral side; similarly control uninjured animals do not show any accumulation of PET signal in the vessels.

From the MR image it can be observed that the vessel wall is thicker for the injured carotid and the contrast is enhanced relative to the uninjured control. Fig. 2C presents a 3D reconstruction of the PET and high resolution MRI data and illustrates how macrophage accumulations can be mapped by MRI to detailed distributions (green regions at arrows) on the vessel wall (purple  =  injured carotid, yellow  =  uninjured contralateral carotid). For example, a ring-shaped lesion (white arrow) was observed, this would be the expected pattern for a highly vulnerable plaque where the macrophages are concentrated in the plaque periphery. The PET signal again covers a larger volume (rendered as an isosurface). This model validates the ability for the multimodal agent to be used for screening by PET followed by high-resolution visualization of the pattern of macrophage distribution by MRI.

**Figure 3:**
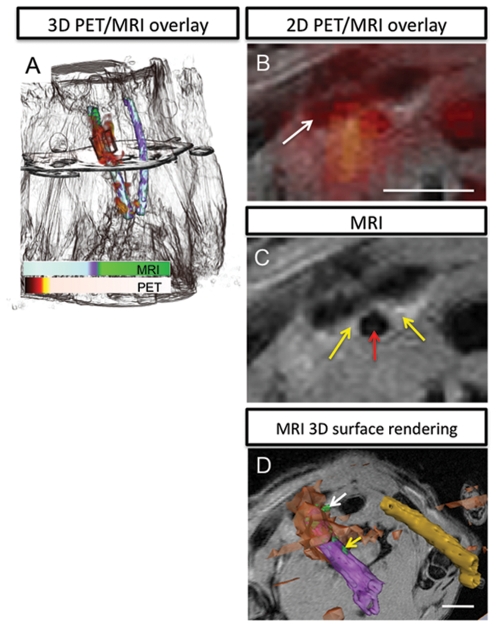
Multimodal macromolecular probes localize to the injured vessel in the rat copper cuff model. (A) Overlay of PET with MRI for nearly whole body 24 h post injection of the PET/T1-MRI probes (head at top, out of field of view). Probe accumulation as monitored by PET is found in a diffuse cloud (red-orange) around the vessel that was clamped by the copper cuff, left (rendered in black and white). The PET and MR signal from vessels was rendered in color using the assignments shown in the scale bar, and MR signal from rest of the body is given in black-and-white for anatomical reference. The plane through the image indicates the position of the image slice given in (B), a view zoomed in around the carotid artery at the superior end of the copper cuff. This coregistered MRI/PET image shows diffuse cloud of PET signal in the region around the injured vessel (clavicle is the dark region indicated by the white arrow) and region of higher MR intensity on the right side of the vessel. scale bar  = 2.5 mm. (C) The MR image from the same plane clearly shows that the vessel (red arrow) has elevated MR contrast in the walls of the vessel (yellow arrows). (D) 3D reconstruction of MRI and PET data in an oblique orientation demonstrates the mapping capabilities for MRI, which is able to identify discrete accumulations of macrophages on the vessel wall. The indicated MR volume (green) at the white arrow is 0.74×0.57×0.46 mm^3^. The volume at the yellow arrow is 0.68×0.60×0.32 mm^3^. Scale bar  = 5 mm. This view is zoomed out from the FOV in panels B and C to include both vessels. Injured carotid artery is purple, increased MR signal intensity relative to vessel background is green, PET signal is orange, and contralateral vessel is gold.

To confirm that labeling of inflammation was independent of injury methodology a second rat model was used that implanted a copper cuff around the right carotid artery (number of animals  = 4). A representative animal is shown in Figure 3. Fig. 3A shows overlaid 3D PET and MR images in the copper cuff model 3 weeks after cuff implantation (Video S4). The PET signal resides over a broad region correlating with the cuffed carotid; MR signal reveals the contours of the animal for anatomical reference, and the position of the cuff and carotid vessels. Overlap of the PET and MRI signals is also apparent in the axial view (Fig. 3B). A hypointense region corresponding to the clavicle is indicated at the white arrow. Fig. 3C shows, in an axial view slice, the increased MRI signal (yellow arrow) from the walls of the right carotid artery (artery at red arrow) in a region superior to the copper cuff, 3 weeks post implantation and 24 hours post injection of ^64^Cu/Gd/TAMRA-mal-BSA. The signal-to-noise (SNR) of the entire vessel wall increased from 10.8 to 17.8 (65±6.8% increase) and the CNR from 0.05 to 2.1 (3800±1800% increase), before and 24 hours post administration of the Gd based agent, respectively. Three-dimensional rendering of the vessels, Fig. 3D, again illustrate how MRI is able to discriminate small accumulations of macrophages on the vessel wall while PET provides highly sensitive detection of injured vessel.

**Figure 4:**
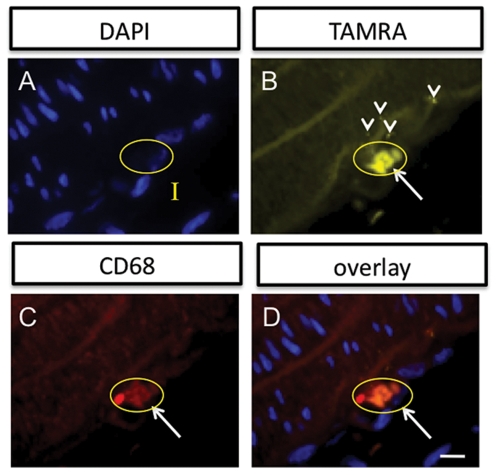
Immunohistochemistry demonstrates that the multimodal probes accumulate in macrophages found in the intimal layer (I) of the injured vessel wall. (A) Cell nuclei stained with DAPI (100X). A single cell is circled in yellow that will be referenced in the other panels. (B) The TAMRA signal from the multimodal agent is found in several locations in the intima. It can be seen in places to be in a punctate patern (arrowheads), suggesting internalization. A pronounced accumulation is shown for the circled cell. (C) Anti-CD68 monoclonal antibody staining also highlights a region in the intima (circled cell at white arrow). (D) Overlay of the three channels, A-C, demonstrates that the TAMRA signal colocalizes with the CD68 positive cell with the nucleus indicated in A, supporting macrophage labeling by the probes. Scale bar  = 25 microns.

To confirm that the imaging probes accumulated in sites of vascular inflammation, immunohistochemistry was performed, using a primary monoclonal antibody against the macrophage marker CD68 along with a secondary antibody conjugated to Dyelight649. A representative field of view at high magnification (100X) is shown in Figure 4. Probe uptake could be tracked using the attached TAMRA dye. DAPI for nuclear staining (Fig. 4A), allowed identification of the intima (I). A single cell in the intima is circled and it also referenced in the rest of the panels in Fig. 4. TAMRA signal (Fig. 4B) showed that the probes accumulated in the intima, particularly in an intense region correlating with the circled cell, and in several punctate spots in the intima. CD68 labeling identified the circled cell to be a macrophage (Fig. 4C). Coregistration of the images labeled for cell nuclei (DAPI), probes (TAMRA), and macrophages (antibody) show that the signals overlap, consistent with uptake of the multimodal probes by macrophages (Fig. 4D).

These results demonstrate the efficacy of combined MRI/PET imaging of plaques in living animal models using a multimodal MRI/PET contrast agent for vascular inflammation. MRI and PET imaging of the probes showed sufficient probe uptake for generation of MRI and PET contrast. Two types of agents were developed to evaluate the ability for each to visualize inflamed lesions in the vasculature. As shown here, both T1 and T2-weighting methods were able to detect relatively small lesions in arteries *in vivo*. In general it was easier to identify regions of signal enhancement and the demarcation of enhanced regions was sharper for the positive contrast agents (T1). This is not unexpected given the mechanisms of contrast enhancement for gadolinium versus iron oxide. Because iron oxide affects signal through a local magnetic field effect, it has lower resolution capability than T1 agents, which affect contrast effect through direct interaction with water protons. In addition, the visual identification of signal increases above background features are typically simpler to interpret than signal decreases. For these reasons, positive contrast agents may be preferable for imaging smaller vessels, where the vessel wall may be only a few voxels thick. The robustness of the method is demonstrated in that localization of the probe to plaques could be observed in several different animal models of vascular inflammation. We envision that these methods could be used as a clinical diagnostic tool wherein PET is used for screening large volumes to identify broad vascular regions that accumulate the macrophage targeted probes. This information is used to guide MR imaging at high resolution to visualize the vessel walls, plaques, and macrophage distribution patterns. Videos are available for this article (Videos S5, S6) that demonstrate how the resulting MR data can be presented to provide a type of noninvasive “endoscopy”. Together the information about macrophage density and distribution can be used to estimate plaque probability to rupture and guide patient management decisions.

Alone, PET can provide a rough location for macrophage rich plaques and allow some quantitation, but typical plaques are much smaller than the resolution limit of PET, so this method provides only an averaged assessment over all plaques in a given volume, specific information about individual plaque vulnerability is not possible. Human plaques at their largest are a few millimeters thick, up to ten millimeters long and disease is often diffuse throughout vessels. At a resolution of 8–16 mm for whole body scans PET alone is unable to reveal plaque structure but will primarily give general localization[36]. At this resolution it would be difficult to correlate the PET signal with individual stenoses or to determine which regions, specifically, are at risk to rupture. PET is also unable to provide information on exactly where in the vessel wall the macrophages reside or their distribution pattern, important criteria for assessing degree of vulnerability. MRI has the resolution to map macrophage distribution in plaques but lacks the sensitivity for screening. For high resolution scans, smaller volumes must be addressed to avoid excessively long scan times but without guidance, it can be challenging to select volumes to focus upon. We believe that cardiovascular imaging of plaques is an ideal application for multimodal imaging where clearly the combination of the imaging methods works synergistically to maximize diagnostic potential from each method.

## Supporting Information

Materials and Methods S1 Extended synthetic and methods information.
(0.09 MB DOC)

Video S1 The aortic valve before administration of probes. Slices above and below the plane of Fig 1B are provided in this video clip. Fig 1B is center slice from the set, sandwiched by three slices on either side. Video moves through the image stack to show other structures in the volume around the valve, including the vena cava (dark circular void appearing on right side near end of video).
(2.22 MB MOV)

Video S2 The aortic valve after administration of probes. Slices above and below the plane of Fig 1C are provided in this video clip. Fig 1C is center slice from the set, sandwiched by three slices on either side. Vena cava becomes visible as above, negative contrast centered around the valve.
(2.40 MB MOV)

Video S3 Multimodal nanoparticle probes are taken up by macrophages in the aortic valve of ligated ApoE^−/−^ mice. Fig 1E three-dimensional view.
(2.93 MB MOV)

Video S4 Multimodal macromolecular probes localize to the injured vessel in the rat copper cuff model. Figure 3A three-dimensional view.
(3.20 MB MOV)

Video S5 Multimodal imaging of the inflamed plaque. Alternate view of Figure 3. Injured vessel is depicted in fuschia. The PET signal, in orange, overlays with a large region of the injured vessel. As the PET signal fades, it becomes apparent from the MR signal (green) that the probe is localizing to discrete regions in the injured vesssel, which represent focal accumulations of macrophages. As the vessel wall signal fades, one can observe that the MRI (green) signal is found through the full thickness of the vessel wall.
(2.26 MB MOV)

Video S6 Noninvasive, PET-guided, MRI endoscopy. Alternate view of Fig 3. With the guidance of PET we were able to identify which region of the vessel to image using MRI. These images can allow us to perform high resolution “noninvasive endoscopy” as shown in the clip below. The point of view travels through the lumen of the vessel, revealing in green the sites of macrophage accumulation (as marked by our probes).
(0.58 MB MOV)
