# Supplementary material for: In Vivo Mapping of Vascular Inflammation Using Multimodal Imaging
Source: PLoS One. 2010 Oct 11;5(10):e13254. doi: 10.1371/journal.pone.0013254 (PMC2952595; doi:10.1371/journal.pone.0013254)
Supplement: Materials and Methods S1 — Extended synthetic and methods information. (0.09 MB DOC) [file pone.0013254.s001.doc]

**Supporting Information**

**Methods**

*Synthesis of copper-64 (^64^Cu) labeled dextran sulfate coated iron oxide nanoparticles:*

General. All chemicals were used as purchased. Ferric chloride hexahydrate (99%) was from Acros Organics (Geel, Belgium); ferrous chloride tetrahydrate (>99%), sodium salt, ethylenediamine (>99.8%), and sodium cyanoborohydride (>95%) were from Fluka (Buchs, Switzerland); dextran (10,095 MW) and epichlorohydrin (>99 %) were from Sigma (St. Louis, MO); ammonium hydroxide solution (28-30%) was from EM Science (Lawrence, KS); periodic acid (reagent grade) and sodium hydroxide were from Fisher Scientific (Waltham, MA); *S*-2-(4-isothiocyanatobenzyl)-1,4,7,10-tetraazacyclododecane-1,4,7,10-tetraacetic acid (*p*-SCN-Bz-DOTA) was from Macrocyclics (Dallas, TX); 5-(and-6)-carboxytetramethylrhodamine, succinimidyl ester (TAMRA, SE) was from Invitrogen (Carlsbad, CA); and cupric-64 chloride solution was from Washington State University (St. Louis, MO), Trace Life Sciences (Denton, TX), or MDS Nordion (Ottawa, Canada). Nanopure water (18.0 MΩ·cm) was from a Barnstead nanopure filtration unit. Routine TLC (50/50 vol/vol, methanol/10% ammonium acetate) was performed on all radiolabed contrast agents to determine the purity (e.g. no free ^64^Cu) of the radiolabeled product (typically >99%).

*Synthesis of copper-64 (^64^Cu) labeled dextran coated iron oxide nanoparticles*

Dextran coated particles were synthesized as described previously*(1,* 2). Briefly, dextran is reduced by stirring an aqueous solution of dextran with sodium borohydride (26 equivalents) at room temperature for 12 h, brought to pH 4.5 with 6N HCl and lyopholized. Nanopure water was bubbled with nitrogen for 1.5 h to de-gas and used throughout the experiment. The FeCl_3_⋅6H_2_O was diluted in de-gassed H_2_O (60.7 mg, 2g/27mL) and a reduced dextran solution (83 mg, 2g/100mL) was added to the Fe^3+^ solution. This solution was bubbled with nitrogen for 40 min while cooling to 4^o^C on an ice bath. Ten minutes before use, FeCl_2_⋅4H_2_O was dissolved in de-gassed H_2_O (30 mg, 1g/27mL) and chilled to 4^o^C, and then added to the reaction mixture. This mixture was then bubbled with nitrogen for an additional 5 min. Chilled (4^o^C) NH_4_OH (0.128 mL) was then added under nitrogen drop-wise with a syringe with rapid magnetic stirring over 2 min. The Fe^3+^/Fe^2+^ ratio was kept at 1.47-1.49, the Fe^3+^/dextran molar ratio was 27.4:1 and the NH_4_OH/Fe^3+^ was kept at 16 molar equivalents for all reactions. The slurry was then heated to 80^o^C and kept at this temperature for 2 h under nitrogen with continuous stirring. The product was cooled to room temperature by removing the heat source and then ultrafiltered (Amicon 8050, MWCO 30 kDa) against ~250 mL nanopure water (6 passes of H_2_O) to remove excess starting material. The purified product was then filtered with 0.2 μm pore membranes to yield a black, translucent solution. Dextran - coated particles were characterized by NMR relaxivity, dynamic light scattering (DLS), and transmission electron microscopy (TEM), as described previously(1).

Next, the dextran coated particles were cross-linked and aminated*(3,* 4). Cross-linked iron oxides (CLIO) were synthesized by adding epichlorohydrin (510 molar equivalents to Fe) to an aqueous solution of dextran coated particles in 2.5M NaOH (0.5 mol NaOH:12 mmol Fe), and stirring at room temp (RT) over night. CLIO was purified by dialysis against nanopure water and no change in particle size was observed by DLS. The CLIO particles were then aminated by addition of ammonium hydroxide (NH_4_OH, 28-30%) in a 10:1 (volume:volume) NH_4_OH/particle solution. This solution was stirred overnight at RT and then purified by dialysis. The purified product was then filtered with 0.2 μm pore membranes to yield a black, translucent solution. Amine termination was then determined by the OPA assay(5), and further characterized by NMR relaxivity, dynamic light scattering (DLS), and transmission electron microscopy (TEM), as described previously(1). No changes in size were noted between CLIO-NH_2_ and the original dextran coated particles.

Radiolabeling of the dextran particles was achieved by first mixing ^64^CuCl_2_ in 10 mM HCl with 1 M triethanolamine acetate to form a ^64^Cu-OAc complex in a 0.1 M TEAA solution with a pH of 7.0 (6 μL, 2.84 mCi ^64^CuOAc used for reaction). Pre-insertion of the metal into the chelator was done by mixing *p*-SCN-Bz-DOTA (0.2 µmol, 10 µL) with the copper-64 acetate solution at 55^o^C for 30 min. The CLIO-NH_2_ nanoparticles (150 μL, 0.1 M TEAA, pH 7, 28.7 µmol Fe) were then reacted with *p*-SCN-Bz-DOTA(^64^Cu) (2.84 mCi) at 55^o^C for 60 min. The nanoparticles were purified by size exclusion chromatography (SEC) on a Sephadex G25 column equilibrated with 0.9% NaCl using centrifugation (2000 rpm, 2 min). This was repeated 3 times to yield CLIO-DOTA(^64^Cu). The activity was then measured with a dose calibrator and the radiolabeling/conjugation yield determined by comparing the activity after purification to that before purification.

*Synthesis of gadolinium/^64^Cu labeled maleylated bovine serum albumin:*

# Copper-64 labeling of maleylated bovine serum albumin (Mal-BSA) was performed as described previously(6). Briefly, a suspension of BSA (Acros Organics, Belgium) was dissolved in 16 mL of 0.1 M of sodium bicarbonate (pH 8.5, 10mg/mL). Next 5-(and-6)-carboxytetramethylrhodamine, succinimidyl ester (TAMRA, SE, Invitrogen, Carlsbad, CA) was dissolved in DMSO (10 mg/mL) and 3.5 equivalents of TAMRA were added to the BSA solution and stirred in the dark at RT for 4 hours. This was purified by gel chromatography on a Sephadex G25 column equilibrated with 0.1 M sodium bicarbonate (pH 8.5) to yield BSA-TAMRA. To this albumin solution a 60 molar excess of *p*-SCN-Bz-DOTA (20 mg/mL in DMSO) was added in 100 µL aliquots with rapid stirring at RT. The pH was monitored continuously during the additions and adjusted by dropwise addition of 1 M sodium bicarbonate (pH 9.0). Once the reaction had reached completion, as shown by no further change in pH, the mixture was dialyzed (50,000 MW pore size, SpectraPor 7) for 12 h against 0.1 M of sodium bicarbonate (pH 8.5) at 4^o^C; yielding 18 DOTA per BSA molecule.

# The product was maleylated using the remaining free lysylamines with maleic anhydride. The reaction of maleic anhydride with amines is competitive with hydrolysis; therefore, 600 molar excess of solid maleic anhydride was added in small quantities to the buffered BSA-TAMRA/(DOTA)_n_ with stirring. The pH was monitored continuously and adjusted by the addition of solid sodium carbonate. The reaction was completed when no further acid was liberated, and the maleylated protein was purified by dialysis (50,000 MW pore size) against 0.1 M sodium citrate (pH 6.6) for no more than 9 h at 4 °C, with minimum of four changes of citrate buffer. The dialyzed maleylated albumin-TAMRA/DOTA solution was then treated with 1.2 molar excess of gadolinium chloride solution (Gd:DOTA) added dropwise while stirring. The pH was maintained between 6.6 and 7.0 using aliquots of 1 M sodium citrate. After the pH had stabilized, the sample was stirred for an additional 24 h at RT. The mal-BSA-TAMRA/(Gd-DOTA)_n_ complex was purified by exhaustive dialysis (50,000 MW, SpectraPor 7) at 4^o^C for 36 h with a minimum of 6 changes of nanopure water. The purified solution of mal-BSA-TAMRA/(Gd-DOTA)_n_ was then lyophilized and stored dessicated at 4 °C. Gadolinium content was determined using ICP-MS and magnetic properties by NMR relaxometry as described previously(6).

Radiolabeling of the MalBSA-TAMRA/(Gd-DOTA)_n_ was achieved as previously described(6). Briefly, ^64^CuCl_2_ (21 µL, 890 µCi in 10 mM HCl) was mixed with (5.25 µL) 1 M triethanolamine acetate (TEAA, pH 7) and incubated at RT for 15 min. Next, 80 µL 0.1M TEAA (pH 7, 0.1 mM) was added to 4 mg lyophilized mal-BSA-TAMRA/(Gd-DOTA)_n_ and this was added to the ^64^Cu-acetate solution, vortexed, and then incubated at 30^o^C for 90 min. The product was then purified three times by SEC using Sephadex G50 equilibrated with 0.9% NaCl by centrifugation (2000 rpm, 2 min).

*In vivo MRI/PET:*

All animal experiments were performed under a protocol approved by the International Animal Care and Use Committee. The temperature inside the coil where the animal was placed was maintained at 37°C, and the ECG and respiration was monitored (MP150, Biopac, Goleta, CA). During imaging the animals were anesthetized by isoflurane inhalation (2% in 100% oxygen, IsoFlo; Abbott Laboratories). Female Sprague Dawley rats (100-125g, Charles River Laboratories, Wilmington, MA) and apoE^-/-^ mice (average 15 months old, Jax West Laboratories, West Sacramento, CA) were used for the experiments.

*Rat imaging*:

Sprague Dawley rats were used as a mechanical injury model of vascular inflammation. Two models were used, (1) a carotid crush injury of the carotid, or (2) implantation of a copper cuff. For the closing the wound site. One, two, or three weeks were allowed for progression of inflammation and recruitment of macrophages before imaging, as it was shown that inflammatory events in the vessel wall after copper cuff implantation progress over three to 42 days(8).

For proof of principle that PET/MR imaging can reveal plaques using scavenger targeted probes, a maleylated-BSA/DOTA(gadolinium/copper-64) contrast was used(6). One week following the crush injury, or at one (n=4), two (n=3), and three (n= carotid crush injury, rats (n=7) were anaesthetized by ketamine/xylazine (88 mg/kg and 2.5 mg/kg) and approximately 1 cm of the common carotid artery was isolated from the carotid sheet and the carotid artery was crushed with tweezers for 10 seconds. One week was allowed for progression of inflammation and recruitment of macrophages, as it has been shown that inflammatory events at the endothelial layer (e.g. expression of inflammatory cell adhesion molecules E-selectin and P-selectin) begin as early as one hour after induction of the injury(7).

*Three dimensional rendering of images:*

For the ligation data set, we produced a three dimensional rendering of the combined MRI and PET data on a commercial 64 bit PC workstation equipped with a commodity graphics processing unit (GeForce GTX 285). The image combines two rendering styles. We display the segmented arteries as opaque surfaces, based on the manual tracing performed in the image analysis stage. To obtain a smooth surface of the segmentation, we re-sample the segmented volume on the vertical direction using cubic interpolation. This method effectively increases the resolution in the vertical dimension to prevent artifacts due to the binary segmentation. To highlight the MR signal on the surface of the arteries, we use a color map from light blue (low MR), through purple (medium) to green (high MR). Smooth color transitions are obtained automatically by the graphic processing unit (GPU), which uses tri-linear interpolation of the MR signal. Tri-linear interpolation smoothly computes the intensity value of a given point by averaging the intensities of the 8 nearest neighbors in the 3D volume. The PET signal is shown as a semi-transparent cloud. This rendering style simulates the result of light going through a semi-transparent attenuating medium. Smooth transitions are also automatically computed by the GPU using tri-linear interpolation.

For the rat copper cuff model, the three-dimensional rendering of MRI/PET overlay is combines three rendering styles. (1) The segmented arteries are also displayed as opaque surfaces, and re-sampled using a cubic interpolation filter, which prevents loss of data, but creates smooth transitions between consecutive slices. To highlight the MR signal on the surface of the arteries, we use a color map from light blue (low MR), through purple (medium) to green (high MR). (2) The PET signal is shown as a semi-transparent cloud. To isolate the PET signal around the vessels, we obtained a distance transform of the injured artery, and highlight only the PET signal that is near the vessel. This distance transform is a derived 3D data set that represents the shortest distance of every point to the surface of the vessel. (3) To depict the anatomical structures, we render the contours of the MRI signal. We do this by highlighting only those regions in the MR image whose gradient is high and orthogonal to the view direction. The effect of this operation is that only silhouette lines are shown, instead of the semi-transparent clouds typical of three-dimensional rendering of MRI data. We apply a similar transformation to the MR signal near the vessels, which allows us to isolate the copper cuff in the injured artery. To provide additional anatomical context, we interleave a data plane, shown as a cross section.

For the copper cuff model, rats (n=4) were anaesthetized by ketamine/xylazine (75-100 mg/kg and 5 mg/kg) and a silicone-copper cuff was implanted around the right carotid artery(8). The copper cuffs were prepared by looping copper wire (0.1 mm) 3 to 4 times around a 1.8 mm mold and embedding in a thin layer silicone. After curing, the cuffs were cut longitudinally and carefully peeled away and sterilized for implantation. The total size of each cuff was about 5 to 6 mm in length and 4 mm in diameter. For implantation, approximately 1.5 cm of the right common carotid artery was isolated from the carotid sheet and the copper cuff carefully place around the vessel before closing the wound site. One, two, or three weeks were allowed for progression of inflammation and recruitment of macrophages before imaging, as it was shown that inflammatory events in the vessel wall after copper cuff implantation progress over three to 42 days(8).

For proof of principle that PET/MR imaging can reveal plaques using scavenger targeted probes, a maleylated-BSA/DOTA(gadolinium/copper-64) contrast was used(6). One week following the crush injury, or at one (n=4), two (n=3), and three (n=2) weeks after implantation of the copper cuff the dual MRI/PET contrast agent was administered; 400-800 μCi of copper was injected via the tail vein. As a control, un-injured Sprague Dawley rats (n=3) were injected with 400-500 μCi of the maleylated-BSA/DOTA(Gd/^64^Cu) via tail vein.

24 hours after contrast agent injection the rats were anesthetized with isoflurane and scanned with a custom built microPETII(9) and then (while under anesthesia) transported to a separate facility for MRI imaging using a 7T BioSpec (Bruker, Billerica, MA).  For the PET imaging, the axial and transaxial FOVs were 4.9 cm and 8.5 cm, with an energy window of 250-750 keV, a timing window of 6 ns, a scan time of 60 minutes, and a fully 3D *maximum a posterioi* (MAP) reconstruction was performed resulting in a spatial resolution of 0.4 x 0.4 x 0.58 mm^3^ for a 128 x128 x 83 matrix*(*10).  For MR imaging, a respiratory gated T1 weighted FLASH sequence was used with TR = 690 ms, TE = 3.7 ms, flip angle = 45^o^, 59 slices, 0.75 mm slice thickness, a FOV = 5.5 x 4.0 cm^2^, and a matrix of 512x256 resulted in an in-plane resolution of 0.107 x 0.156 mm^2^.  Three fiducial markers (glass bulbs filled with an aqueous solution of ~0.5 µCi ^64^Cu each) were postioned on the animal and used as land markers to co-register the MRI and PET data. 

*Mouse imaging*:

ApoE^-/-^ mice (n=3), average age 13 weeks, underwent the carotid artery ligation and after ligation of the right carotid artery were placed on a high cholesterol diet (20.1% fat, 1.25% cholesterol Harlan Teklad, TD.02028) for two weeks prior to imaging using separate PET and MRI scanners; 24 hours after contrast agent injection the mice were anesthetized with isoflurane and scanned with a custom built microPETII (9) and then (while under anesthesia) transported to a separate facility for MRI imaging using a 7T BioSpec (Bruker, Billerica, MA).  For the PET imaging, the axial and transaxial FOVs were 4.9 cm and 8.5 cm, with an energy window of 250-750 keV, a timing window of 6 ns, a scan time of 60 minutes, and a fully 3D *maximum a posterioi* (MAP) reconstruction was performed resulting in a spatial resolution of 0.4 x 0.4 x 0.58 mm^3^ for a 128 x128 x 83 matrix*(*10).  For MR imaging, a respiratory gated T2* weighted FLASH sequence was used with TR = 540 ms, TE = 5 ms, flip angle = 30^o^, 40 slices, 0.5 mm slice thickness, a FOV = 3.5 x 3.5 cm^2^, and a matrix of 256x256 resulted in an in-plane resolution of 0.137 x 0.137 mm^2^.  Three fiducial markers (glass bulbs filled with an aqueous solution of ~0.5 µCi ^64^Cu each) were positioned on the animal and used as land markers to co-register the MRI and PET data.

*Image Analysis*:

For both rat and mouse image data sets, MRI and PET data were analyzed on a commercial 64bit PC workstation. The MRI and PET data coregistered and tridimensional (3D) volumes corresponding to probe uptake (PET) and vasculature (MRI) were traced manually in the axial, coronal, and sagittal planes using Amira 5 (Mercury Computer Systems, Visage Imaging, Carlsbad, CA). Alternatively, the MRI and PET data was exported into Amide version 0.8.22 (Medical Data Image Analyzer) and co-registered. Signal to noise ratio (SNR) and contrast to noise ratio (CNR) of the raw MRI and PET data were determined using ImageJ (National Institutes of Health, version 1.38x). For the MRI data, CNR was defined as abs[Signal_injury_-Signal_tissue_]/Signal_noise_, where the absolute value of the signals is taken to obtain a positive CNR. In the CNR measurements the ROI used was the same size for all areas and the normal tissue signal used for contrast determination was the adjacent vessel tissue to the probe uptake.

*Three dimensional rendering of images:*

For the ligation data set, we produced a three dimensional rendering of the combined MRI and PET data on a commercial 64 bit PC workstation equipped with a commodity graphics processing unit (GeForce GTX 285). The image combines two rendering styles. We display the segmented arteries as opaque surfaces, based on the manual tracing performed in the image analysis stage. To obtain a smooth surface of the segmentation, we re-sample the segmented volume on the vertical direction using cubic interpolation. This method effectively increases the resolution in the vertical dimension to prevent artifacts due to the binary segmentation. To highlight the MR signal on the surface of the arteries, we use a color map from light blue (low MR), through purple (medium) to green (high MR). Smooth color transitions are obtained automatically by the graphic processing unit (GPU), which uses tri-linear interpolation of the MR signal. Tri-linear interpolation smoothly computes the intensity value of a given point by averaging the intensities of the 8 nearest neighbors in the 3D volume. The PET signal is shown as a semi-transparent cloud. This rendering style simulates the result of light going through a semi-transparent attenuating medium. Smooth transitions are also automatically computed by the GPU using tri-linear interpolation.

For the rat copper cuff model, the three-dimensional rendering of MRI/PET overlay is combines three rendering styles. (1) The segmented arteries are also displayed as opaque surfaces, and re-sampled using a cubic interpolation filter, which prevents loss of data, but creates smooth transitions between consecutive slices. To highlight the MR signal on the surface of the arteries, we use a color map from light blue (low MR), through purple (medium) to green (high MR). (2) The PET signal is shown as a semi-transparent cloud. To isolate the PET signal around the vessels, we obtained a distance transform of the injured artery, and highlight only the PET signal that is near the vessel. This distance transform is a derived 3D data set that represents the shortest distance of every point to the surface of the vessel. (3) To depict the anatomical structures, we render the contours of the MRI signal. We do this by highlighting only those regions in the MR image whose gradient is high and orthogonal to the view direction. The effect of this operation is that only silhouette lines are shown, instead of the semi-transparent clouds typical of three-dimensional rendering of MRI data. We apply a similar transformation to the MR signal near the vessels, which allows us to isolate the copper cuff in the injured artery. To provide additional anatomical context, we interleave a data plane, shown as a cross section.

*Histology:*

To confirm the presence of macrophages, tissue samples were prepared for immunohistochemistry following the imaging experiments. After the 24 hour imaging point, the animals were euthanized and the vasculature was rinsed with heparinized saline via cardiac perfusion. The vessels were then isolated and placed in 10% buffered formalin containing 0.2% trypsin (for membrane permiabilization, necessary for antibody treatment). Note that for the copper cuff the cuff was carefully removed from the artery before removing the artery from the animal and placing in fixative. Tissue was put into fixative for 8 hours at 4^o^C before transferring to 70% ethanol, and stored in ethanol at 4^o^C until all radioactive decayed before embedding. Samples were embedded in paraffin and 4 micron thick slices were obtained. Samples were then deparaffinized, rehydrated, and then antigen retrieval was performed by heating the samples in 10 mM citrate buffer (pH 6) for 1 hour in a steam chamber, then incubating the samples (still in the citrate buffer) in at 4^o^C overnight.

After antigen retrieval, endogenous enzyme activity was blocked with peroxide (10 min, RT, 0.3% H_2_O_2_/methanol), followed by non-specific blocking with BSA (1.2mg/mL, 4^o^C overnight). Samples were then incubated with the macrophage specific primary antibody anti-CD68 (Serotec, Oxford, UK) at a 1:100 dilution overnight at 4^o^C (rat anti-mouse CD68 for apoE^-/-^ mice or mouse anti-rat CD68 for the copper cuff rats). For the rats, a goat anti-mouse secondary antibody coupled to dyelight647 (Serotec) was then incubated at a 1:100 dilution for 1.5 hours at RT and counterstained with DAPI (300nM, 5 min). Then a glass cover slip was mounted with Vectashield H-1000 mounting medium (Vector Laboratory, Burlingame, CA), and samples were imaged by fluorescent microscopy on a Nikon Eclipse TE2000-S scope using a 100x (Nikon PlanApo, NA1.4) oil immersion lens. A Nikon mercury arc lamp was used to excite samples with chroma dichroic filter sets for DAPI (Ca^2+^ 380nm excitation pass), BSA-TAMRA (FITC/TRITC bandpass), and goat anti-mouse IgG-Dyelight647 (FITC/CY5 bandpass).

For the apoE^-/-^ mice a rabbit anti-rat secondary antibody coupled to horse radish peroxidase (HRP, Serotec) was incubated with the samples at a 1:100 dilution for 1.5 hours at RT. The bound antibody was visualized by counterstaining with diaminobenzidine (DAB); samples were incubatd for 10 min at RT in a glass jar with 200 ml of Tris-HCl buffer (pH, 7.5) containing 40 mg DAB and 34 mL 30% H_2_O_2_. After thurough washing to remove excess DAB, the slides were counterstained for iron using the Perl’s Prussian blue stain. A coverslip was mounted (Vectashield) and images were acquired with a Canon Cybershot MPEG Movie EX 5.0 megapixel camera with a Scopetronix Maxview 40 plus adapter attached to a Olympus BX51 scope using a 10x (UPlanFluor, 0.3 NA) or 60x (PlanApo, 1.4 NA) oil immersion objective.

1. B. R. Jarrett, M. Frendo, J. Vogan, A. Y. Louie, *Nanotechnology* **18**, 035603 (Jan, 2007).

2. B. R. Jarrett, B. Gustafsson, D. L. Kukis, A. Y. Louie, *Bioconjugate Chemistry* **19**, 1496 (Jul, 2008).

3. S. Palmacci, L. Josephson. (Advanced Magnetics, Inc. (Cambridge, MA), United States of America, 1993), pp. 1-18.

4. G. von Maltzahn *et al.*, *Bioconjugate Chemistry* **19**, 1570 (Aug, 2008).

5. J. R. Benson, P. E. Hare, *Proc Natl Acad Sci U S A* **72**, 619 (Feb, 1975).

6. B. Gustafsson, S. Youens, A. Y. Louie, *Bioconjugate Chemistry* **17**, 538 (Mar-Apr, 2006).

7. P. Zoldhelyi *et al.*, *American Journal of Physiology-Heart and Circulatory Physiology* **279**, H3065 (Dec, 2000).

8. W. Volker *et al.*, *Atherosclerosis* **130**, 29 (Apr, 1997).

9. Y. C. Tai *et al.*, *Physics in Medicine and Biology* **48**, 1519 (Jun, 2003).

10. Y. F. Yang *et al.*, *Physics in Medicine and Biology* **49**, 2527 (Jun, 2004).

11. C. Catana *et al.*, *Proceedings of the National Academy of Sciences of the United States of America* **105**, 3705 (Mar, 2008).
